# A case series of ischemic stroke with coronavirus disease 2019 in two Egyptian centers

**DOI:** 10.1186/s41983-020-00259-z

**Published:** 2020-12-24

**Authors:** Nevine El Nahas, Tamer Roushdy, Eman Hamid, Sherien Farag, Hossam Shokri, Mai Fathy, Eman Abushady, Ahmed Hazzou, Hany Aref

**Affiliations:** Neurology Department, Faculty of Medicine, Ain Shams University, Cairo, Egypt

**Keywords:** Stroke, Egypt, COVID-19, SARS-CoV-2

## Abstract

**Background:**

Severe acute respiratory syndrome coronavirus 2 (SARS-CoV-2) is a novel virus that has been reported to have various neurological manifestations. Cerebrovascular disorders have been encountered as a coronavirus disease 2019 (COVID-19) presentation in our center during the pandemic.

**Case presentation:**

We are presenting 10 cases with cerebrovascular manifestations after having COVID-19 few days prior to stroke.

**Conclusion:**

Cerebrovascular manifestations can occur in association with COVID-19 and may have significant implications on prognosis and management.

## Background

COVID-19 has been declared by the WHO as a pandemic in March 2020. Since then, here have been reports of patients with COVID-19 presenting solely with neurological manifestations [1] or in addition to the classic respiratory symptoms [2]. In a recent study, 36.4% of patients had neurological manifestations, such as headache, dizziness, acute cerebrovascular diseases, and impaired consciousness [3].

The first COVID-19 case announced in Egypt was on 15 February. At this point, a modified pathway for dealing with suspected cases presenting to ER with stroke and signs of viremia (fever, malaise, and cough) was implemented, in which a CT brain with or without CT angiography according to clinical suspicion of proximal occlusion and CT chest are ordered beside labs and nasopharyngeal swab. Patient is then managed according to guidelines for acute stroke arriving in window of thrombolysis and/or thrombectomy and is admitted to isolation unit, and on obtaining PCR swab results (approximately within 48 h), the patient is to be managed at the stroke center or transferred to quarantine hospital with initial stroke management recommendations [4]. In our stroke center, we started seeing COVID patients presenting with acute ischemic stroke (AIS) on 28 April.

In this case series, we are presenting 10 cases with AIS after having COVID-19 few days prior to stroke. They were admitted to Ain Shams University Hospital Stroke Centers. COVID-19 symptoms ranged from fever to dry cough, shortness of breath, and myalgia. All patients were proven to be COVID-19 positive by polymerase chain reaction (PCR) (Table 1).

**Table 1:** Clinical characteristics of the patients

	Age	Gender	Medical history	Stroke presentation	NIHSS on admission and post-r-tPA	COVID-19 severity	Lab results (all patients are COVID-19 PCR positive)			Imaging (brain and chest)	Treatment received	Outcome and mRS
							CBC ^**a**^	Acute phase reactants^**b**^ (CRP, ESR, Ferritin, LDH)	D-dimer^**c**^			
**Patient 1**	65	Male	o Ex-smoker o HTN o AF o ISH o BA	**OTD** 1.5 h o RT hemiparesis o RT hemihypesthesia o RT UMN facial o Global aphasia	**12 and 9**	Moderate	**HB** 16.9 g/dl, **WBC** 12.6; **Lymph.** 1.76, **neutrophil** 10.27, **platele**t 340	**CRP** 315 ↑, **ESR** 10, **ferritin** 113	1.62 ↑	**CT brain:** baseline: free, **MRI:** MCA with hemorrhagic transformation **CT chest**: multiple unilateral right lower lung lobe ground glass appearance and small peripheral consolidation patches of intermediate probability for COVID	**For stroke**: r-tPA DTN 25 min **For COVID-19:** oseltamivir, azithromycin, LMWH	Transfer to isolation unit Discharged after 20 days **mRS** 4
**Patient 2**	57	Male	o ISH o HF o DM	**OTD** 3 h o LT hemiparesis o LT hemihypesthesia o LT UMN facial o Partial gaze o Dysarthria	11 and 7	Severe	**HB** 15 g/dl, **WBC** 7.2, **Lymph.** 0.91↓, **neutrophil** 5.44, **platelet** 297	**CRP** 410↑, **ESR** 45	2.35↑	**CT brain**: baseline: free **X-ray chest:** bilateral ground glass opacities peripheral and central with high probability of COVID	**For stroke**: r-tPA DTN 33 min **For COVID-19:** azithromycin, 40 mg steroids	Desaturation and put on oxygen 2 L. ICU admission and ventilated for 1 day then died. **mRS** 6
**Patient 3**	56	Male	None	**OTD** 2.5 h o LT hemiparesis o LT hemihypesthesia o Ataxia o Dysarthria	7 and 4	Moderate	**HB** 16.1 g/dl, **WBC** 7.5, **Lymph.** 0.87↓, **neutrophil** 5.96, **platelet** 223	**CRP** 1071↑, **ESR** 40	2.16↑	**CT brain**: baseline: free **CT chest:** bilateral scattered central and peripheral ground glass opacities with high probability of COVID	**For stroke**: r-tPA DTN 20 min **For COVID-19:** LMWH 60 IU, azithromycin, 40 mg steroids	Transfer to ICU isolation unit **mRS** 2
**Patient 4**	52	Male	o Ex-smoker o ISH, o History of pulmonary edema o DM	**OTD** 1 h o RT hemiparesis o RT hemihypesthesia o RT UMN facial o Global aphasia	12 and 5	Moderate	**HB** 14.5 g/dl, **WBC** 7.5, **Lymph.** 1.17, **neutrophil** 5.52, **platelet** 228	**CRP** 306↑, **ESR** 50	1.95↑	**CT brain**: baseline: free **Follow-up:** MCA territorial infarction **CT chest:** bilateral central areas of ground glass with mild pulmonary venous congestion with minimal pleural effusion and tiny calcified pleural plagues	**For stroke**: r-tPA DTN 30 min **For COVID-19:** azithromycin, LMWH, 40 mg steroids	Transfer to isolation unit, discharged later on **mRS** 2
**Patient 5**	74	Female	o DM o HTN	**OTD** 3 h o RT hemiparesis o RT hemihypesthesia o RT UMN facial o Dysarthria o Inattention o Diabetic ketosis (RBG 500 placed on insulin pump)	12 and 8	Mild	**HB** 11.5 g/dl, **WBC** 8.6, **Lymph** 1.07, **neutrophil** 6.37, **platelet** 312	**CRP** 120↑, **ESR** 70, **LDH** 234↑	2.44↑	**CT brain**: baseline: free **CT chest:** bilateral diffuse basal ground glass appearance and interseptal thickening—indeterminate for COVID	**For stroke**: r-tPA DTN 30 min **For COVID-19:** azithromycin, oseltamivir	Transferred to isolation unit and discharged home after stabilization **mRS** 2
**Patient 6**	68	Female	o HTN	**OTD** 3 h o LT hemiparesis o LT hemihypesthesia o LT UMN facial o Gaze	14 and 6	Mild	**HB** 12.5 g/dl, **WBC** 4.3, **Lymph.** 1.46, **neutrophil** 2.51, **platelet** 232	**CRP** 164↑, **ESR** 45, **ferritin** 590.6↑	3.36↑	**CT brain**: baseline: free **Follow-up:** RT parietal and frontal MCA infarction **CT chest:** bilateral central patchy areas of ground glass opacity—intermediate probability of Covid-19	**For stroke**: r-tPA DTN 35 min **For COVID-19:** azithromycin, oseltamivir, full dose LMWH	Transfer to isolation room within department and then to isolation unit—much improved. **mRS** 2
**Patient 7**	59	Male	o DM	**OTD** 3 h o RT hemiparesis o RT hemihypesthesia o RT UMN facial o Global aphasia	17 and 15	Severe	**HB** 12.5 g/dl, **WBC** 10.7, **Lymph.** 0.8↓, **neutrophil** 11, **platelet** 238	**CRP** 302.5↑, **ESR** 55, **ferritin** 1300↑	1.91↑	**CT brain:** baseline: free **Follow-up:** left MCA infarction **CT chest:** bilateral basal ground glass	**For stroke**: r-tPA DTN 35 min	Desaturated on room air (So2 66%), intubated and ventilated with ICU admission and died the next day **mRS** 6
**Patient 8**	62	Female	o AF o ISH o DM o HTN	**OTD** 7 h o LT hemiparesis o LT hemihypesthesia o LT UMN facial	10	Moderate	**HB** 11 g/dl, **WBC** 8.7, **Lymph.** 1.33, **neutrophil** 6.25, **platelet** 216	**CRP** 78↑, **ESR** 58	1.85↑	**MRI brain:** right external watershed area **CT chest:** 2 small areas with ground glass appearance, mild right and minimal left-sided pleural effusion, low to intermediate left-sided pleural effusion	**For COVID-19:** chloroquine, oseltamivir, azithromycin, full dose anticoagulation	Transfer to isolation unit and was stable for 12 days **mRS** 4 Then sudden deterioration with hemorrhagic transformation GCS 3
**Patient 9**	37	Male	o Smoking o HTN	**OTD** 24 h o LT hemiparesis o LT UMN facial o Dysarthria	10	Moderate	**HB** 12.1 g/dl, **WBC** 9.55, **Lymph.** 2.36**, neutrophil** 6.26, **platelet** 255	**CRP** 70↑, **ESR** 63	2.41↑	**MRI brain:** RT basal ganglion large ischemic infarction **X-ray chest:** free except for increased bronchovascular markings	**For stroke and COVID-19:** antiplatelet then anticoagulation	Discharged and much improved **mRS** 1
**Patient 10**	36	Male	None	**OTD** 12 h o Expressive dysphasia	4	Mild	**HB** 13.1 g/dl, **WBC** 5.92, **Lymph.** 2.03**, neutrophil** 2.92, **platelet** 273	**CRP** 38.9↑, **ESR** 55, **ferritin** 584.1↑		**MRI brain:** LT insular Fronto-parietal. Left cerebellar subacute infarction **CT chest:** bilateral multilobar multifocal variable-sized patchy and wedge shape ground glass opacities, bilateral lower lobe consolidations shows predominant peripheral distribution—highly suspicious of COVID	**For stroke and COVID-19:** antiplatelet then anticoagulant	Stable despite suffering acute myocardial infarction after stroke by few days. **mRS** 0

*HTN* hypertension, *DM* diabetes mellitus, *ISH* ischemic heart, *AF* atrial fibrillation, *BA* bronchial asthma, *UMN* upper motor neuron, *HB* hemoglobin, *WBC* white blood cells, *Lymph.* lymphocytes, *CRP* C-reactive protein, *ESR* erythrocyte sedimentation rate, *LDH* lactate dehydrogenase, *mRS* modified Rankin Score, *MCA* middle cerebral artery, *RT* right, *LT* left, *OTD* onset to door, *DTN* door to needle, *NIHSS* National Institute of Health Stroke Scale, *LMWH* low molecular weight heparin, *GCS* Glasgow coma scale

^a^HB = normal value 13–17 g/dl, WBC = normal value 4000–10,000, neutrophils = normal value 2000–7000, lymphocytes = normal value 1000–3000, 20–40%, platelets = normal value 250,000–450,000

^b^CRP normal value up to 6 mg/l, ESR normal value up 2–20 mm/h, ferritin = normal value 13–150 ng/l, LDH normal value 140–270 IU/l

^c^D-dimer = normal value up to (0.55 ug/l)

## Cases presentation

### Patient 1

A 65-year-old male, ex-smoker, hypertensive, with ischemic heart disease (IHD), atrial fibrillation (AF), and bronchial asthma since childhood, presented 2 days after COVID-19 symptoms. AIS onset-to-door time (ODT) was 1 and a half hour of right hemiparesis and global aphasia (National Institutes of Health Stroke Scale (NIHSS) 12). Investigations showed elevated D-dimer and CRP; computed tomography (CT) chest showed intermediate probability of COVID-19 and normal CT brain. Intravenous thrombolysis (IV r-tPA) was given with door-to-needle time (DNT) of 25 min. Two hours NIHSS was 9, and follow-up magnetic resonance imaging (MRI) (Philips 1.5 Tesla, Germany) showed middle cerebral artery (MCA) M1 territory recent infarction with hemorrhagic transformation. Patient was admitted to isolation unit, then transferred to quarantine hospital and discharged after 20 days with modified Rankin score (mRS) of 4.

### Patient 2

A 57-year-old male, with IHD, heart failure with EF 37%, and diabetes, presented 4 days following COVID-19 symptoms. ODT was 3 h of left hemiparesis and partial gaze palsy (NIHSS 11). Investigations showed grade I lymphopenia and elevated D-dimer, CRP, and ESR; X-ray chest showed high probability of COVID-19, and CT brain was normal. r-tPA was initiated with DNT of 33 min. Two hours NIHSS was 7. Patient showed signs of desaturation and was mechanically ventilated and died 3 days from admission.

### Patient 3

A 56-year-old male patient, with irrelevant medical history, presented 2 days following COVID-19 symptoms. ODT was 2 and a half hours of left ataxic hemiparesis and dysarthria (NIHSS 7). Investigations showed grade I lymphopenia and elevated D-dimer, CRP, and ESR; CT chest showed high probability of COVID-19 and normal CT brain. r-tPA was given with DNT of 20 min; 2 h NIHSS was 4. Patient was admitted to isolation ward then to quarantine hospital with mRS 2.

### Patient 4

A 52-year-old male, ex-smoker, diabetic, IHD, with history of pulmonary edema, EF 38%, presented 3 days following COVID-19 symptoms. ODT was 1 h of right hemiparesis and global aphasia (NIHSS 12). Investigations showed relative lymphopenia and elevated D-dimer, CRP, and ESR; CT chest showed low probability of COVID, and CT brain was normal. Patient received r-tPA with DNT of 30 min. Two hours NIHSS was 5; follow-up brain imaging showed territorial MCA infarction. Patient was admitted to isolation unit and on obtaining PCR results transferred to quarantine hospital and discharged with mRS 2.

### Patient 5

A 74-year-old female, diabetic and hypertensive, presented 3 days following COVID-19 symptoms. ODT was 3 h of right hemiparesis and diabetic ketosis for which insulin pump was used (NIHSS 12). Investigations showed relative lymphopenia, elevated D-dimer, and inflammatory markers; CT chest was indeterminate for COVID-1, and CT brain was normal. r-tPA was given with DNT of 30 min; 2 h NIHSS was 8. Patient was transferred to isolation unit then to quarantine hospital and discharged home with mRS 2.

### Patient 6

A 68-year-old female, hypertensive, presented 2 days following COVID-19 symptoms. ODT was 3 h of left hemiparesis and gaze (NIHSS 14). Investigations showed elevated D-dimer, ferritin, and inflammatory markers; CT chest showed intermediate probability of COVID-19, and CT brain was normal. r-tPA was given with DNT of 35 min; 2 h NIHSS was 6. Follow-up MRI showed right MCA territory infarction; patient was transferred to isolation unit then to quarantine hospital with mRS 2.

### Patient 7

A 59-year-old male, diabetic, presented 2 days following COVID-19 symptoms. ODT was 3 h of right hemiparesis and global aphasia (NIHSS 17). Investigations showed grade I lymphopenia, elevated D-dimer, ferritin, and inflammatory markers; CT chest showed intermediate probability of COVID-19; CT brain was normal. He received r-tPA with DNT of 35 min; 2 h NIHSS was 15. MRI brain showed recent left MCA infarction. Patient developed desaturation on room air, SO_2_ 66%, intubated and ventilated, and died within 3 days of admission.

### Patient 8

**Fig. 1:**
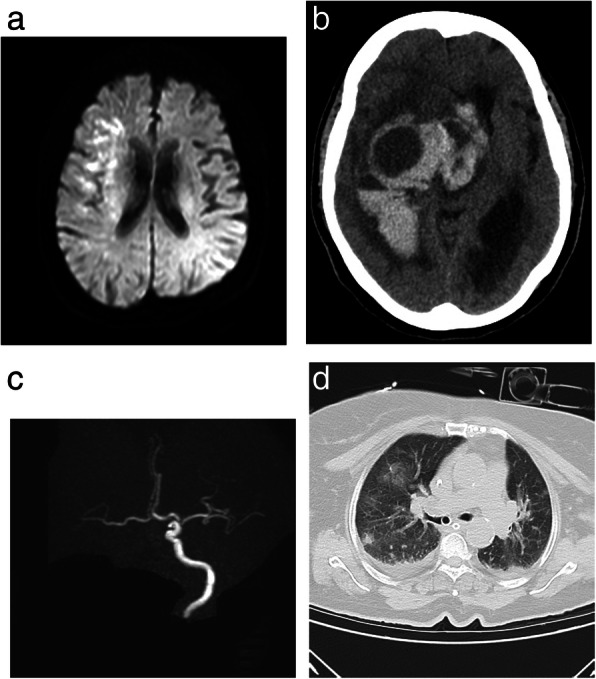
Patient 8, **a** MRI DWI with right external watershed infarction, **b** complete occlusion of right internal carotid artery, **c** follow-up CT brain after sudden deterioration on day 12 showing hemorrhagic transformation, interventricular extension, and midline shift, **d** CT chest showed 2 small areas of ground glass appearance, mild right and minimal left-sided pleural effusion, low to intermediate left-sided pleural effusion

A 62-year-old female, diabetic, hypertensive, with IHD and AF, presented 1 day following COVID-19 symptoms with ODT of 7 h of left hemiparesis (NIHSS 10). Investigations showed relative lymphopenia, elevated D-dimer and inflammatory markers, and bilateral basal ground glass imaging on CT chest; MRI brain revealed right external watershed infarction. Patient condition was stable over 12 days with mRS 4, then suddenly deteriorated with Glasgow coma scale 3; CT brain revealed hemorrhagic transformation, interventricular extension, and midline shift (Fig. 1). The patient is still on mechanical ventilator.

### Patient 9

A 37-year-old male, hypertensive and smoker, presented 1 day following COVID-19 symptoms with ODT of 1 day with left hemiparesis and dysarthria (NIHSS 10). Investigations showed elevated D-dimer and inflammatory markers, X-ray chest was normal, and brain imaging revealed right basal ganglionic infarction; patient was admitted to isolation unit with initiation of antiplatelet and anticoagulant, with much improvement and discharged with mRS 1.

### Patient 10

**Fig. 2:**
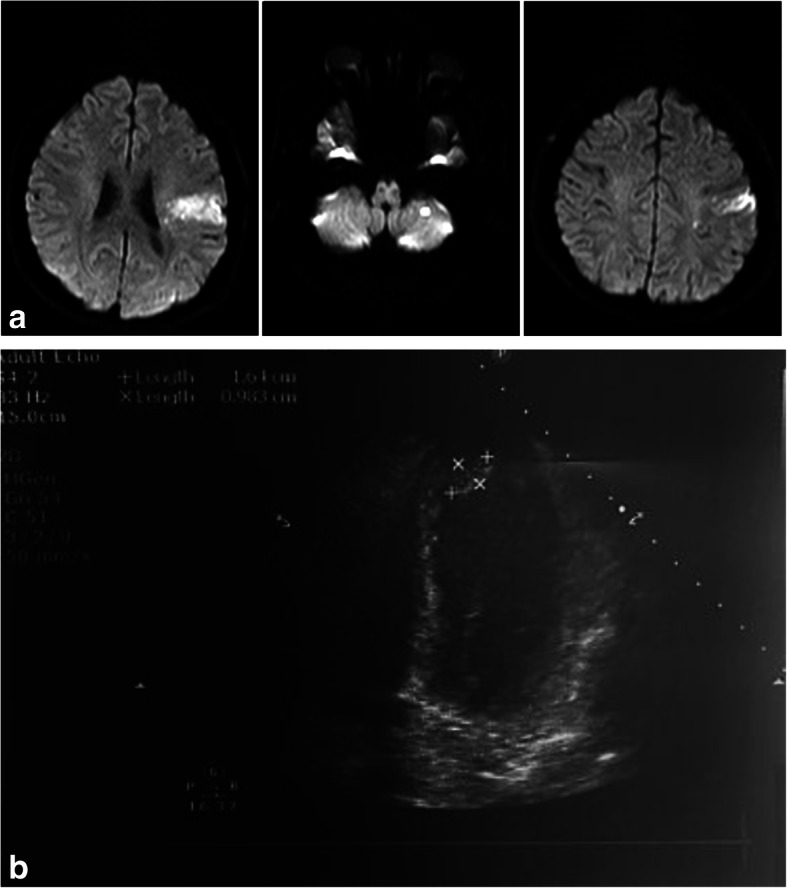
**a** MRI DWI of patient 10 with left insular and cerebellar infarction. **b** ECHO of patient 10 with fresh apical thrombus measuring 12 × 16 mm, EF 35%, akinetic all apical segment, mid-anterior wall, mid-anterior septum, and mid-posterior septum

A 36-year-old male, with irrelevant medical history, presented 7 days following COVID-19 symptoms with ODT of 12 h of aphasia (NIHSS 4). Investigations showed elevated ferritin and inflammatory markers; chest imaging showed high probability of COVID-19; brain imaging showed left insular and left cerebellar subacute infarction. Patient received antiplatelet and was stable with mRS 0, few days later developed acute myocardial infarction; transthoracic echocardiography (Vivid E9 machine, General Electric, Vingmed Ultrasound, Horten, Norway) revealed fresh apical thrombus with elevated troponin (0.321 ng/ml—normal range < 0.014 ng/ml), and anticoagulation was initiated (Fig. 2). The patient became stable and was discharged.

## Discussion

All cases presented with AIS ranging from 1 to 7 days after onset of viremia symptoms which included fever, dyspnea, fatigue, and/or myalgia. CT chest was suggestive of COVID-19 criteria; D-dimer was mildly elevated, with moderately to markedly increased CRP and ESR. Lymphocytic count ranged from normal to relative or grade I lymphopenia. Similar findings were reported by other groups [5–7]. All cases were confirmed with a positive PCR within 48 h of presentation.

Seven patients received thrombolytic therapy, and the rest either anticoagulant or antiplatelet therapy. Six patients improved as regards stroke with mRS ranging from 0 to 4.

Four out of ten patients (patients 2, 7, 8, and 10) developed complications. Only patient 8 had a neurological cause for deterioration, while patients 2 and 7 had sudden desaturation and eventually died. Patient 10 reached mRS = 0, then had a myocardial infarction, but fortunately, he recovered and was discharged. This agrees with what has been suggested that AIS might be associated with severe form of COVID-19 [8].

The controversy whether COVID-19 can cause thrombosis or hemorrhage still holds, as seen in patient 8 who developed hemorrhagic transformation of her infarction. Although patients with increased D-dimer can suffer from thrombotic events, yet hemorrhage due to prolonged prothrombin time might occur [6].

It is worth mentioning that cases 3 and 10 had no risk factors for stroke; in addition, case 10 was only 36 years old yet developed myocardial infarction few days after AIS. This raises the possibility of occurrence of an inflammatory response termed “immunothrombosis” in such cases [9–11]. However, still one cannot overlook the detrimental effect of hyperthermia, dehydration, and hypoxia in the presence of viral endothelial invasion through ACE receptors that are abundant in endothelial cells [5, 12, 13]. It is also reported that COVID-19 has a role in developing diffuse vasculopathy and vasculitis through inflammatory cell infiltration, endothelial cell damage, and endotheliopathy. All these factors can contribute to platelet aggregation and thrombosis [5].

Out of the current case series, we have the following recommendations: COVID-19 has pulmonary besides extrapulmonary manifestations, and stroke is one of such manifestations, so triaging patients in ER with a proper baseline investigations as CT chest and nasopharyngeal swab can detect hidden COVID-19 cases that ought to be isolated and at the same time managed as stroke guidelines recommend based on onset to door. In our presented cases D-dimer was mildly elevated and lymphocytic count was showing grade I lymphopenia in some but not all cases so it is worth mentioning that other supportive criteria for COVID-19 like symptomatology, CT chest, and PCR are of greater value than labs.

## Conclusion

Whether a cause or a coincidence, COVID-19 with stroke has been treated by r-tPA, anticoagulants, and/or antiplatelets. Patient 8 developed intracerebral hemorrhage after receiving anticoagulation, while case 10 developed another thrombotic event despite receiving anticoagulant and antiplatelet. So in order to achieve the best outcome and avoid complications, guidelines for the timing and dosing of medications that guard against immunothrombosis need further elaboration.

## Data Availability

The corresponding author takes full responsibility for the data, has full access to all of the data, and has the right to publish any and all data separate and apart from any sponsor.

## Abbreviations

COVID-19: Coronavirus disease 2019
AIS: Acute ischemic stroke
PCR: Polymerase chain reaction
IHD: Ischemic heart disease
AF: Atrial fibrillation
ODT: Onset-to-door time
MRI: Magnetic resonance imaging
MCA: Middle cerebral artery
NIHSS: National Institutes of Health Stroke Scale
CT: Computed tomography
IV r-tPA: Intravenous thrombolysis
DNT: Door-to-needle time
mRS: Modified Rankin score
